# Cold adapted *Pseudomonas*: ecology to biotechnology

**DOI:** 10.3389/fmicb.2023.1218708

**Published:** 2023-07-17

**Authors:** Mansi Chauhan, Ayushi Kimothi, Avinash Sharma, Anita Pandey

**Affiliations:** ^1^Department of Microbiology, Graphic Era (Deemed to be University), Dehradun, Uttarakhand, India; ^2^National Centre for Cell Science, Pune, Maharashtra, India; ^3^Department of Biotechnology, Graphic Era (Deemed to be University), Dehradun, Uttarakhand, India

**Keywords:** *Pseudomonas*, psychrophiles, adaptation, ecological processes, biotechnological applications

## Abstract

The cold adapted microorganisms, psychrophiles/psychrotolerants, go through several modifications at cellular and biochemical levels to alleviate the influence of low temperature stress conditions. The low temperature environments depend on these cold adapted microorganisms for various ecological processes. The ability of the microorganisms to function in cold environments depends on the strategies directly associated with cell metabolism, physicochemical constrains, and stress factors. *Pseudomonas* is one among such group of microorganisms which is predominant in cold environments with a wide range of ecological and biotechnological applications. Bioformulations of *Pseudomonas* spp., possessing plant growth promotion and biocontrol abilities for application under low temperature environments, are well documented. Further, recent advances in high throughput sequencing provide essential information regarding the prevalence of *Pseudomonas* in rhizospheres and their role in plant health. Cold adapted species of *Pseudomonas* are also getting recognition for their potential in biodegradation and bioremediation of environmental contaminants. Production of enzymes and bioactive compounds (primarily as an adaptation mechanism) gives way to their applications in various industries. Exopolysaccharides and various biotechnologically important enzymes, produced by cold adapted species of *Pseudomonas*, are making their way in food, textiles, and pharmaceuticals. The present review, therefore, aims to summarize the functional versatility of *Pseudomonas* with particular reference to its peculiarities along with the ecological and biotechnological applications.

## Introduction

The earth is dominated by a large portion of both terrestrial as well as aquatic low temperature environments. The Polar and Alpine region occupies 25% of the world’s terrestrial area. While 30% of the Earth’s landscape is covered with glaciers, permafrost, snow and ice (Deming, 2002; Yadav et al., 2015c), making them biodiversity hotspots and constitute a separate discipline known as “cryosphere.” The oceans are one of the essential microbiomes consisting of unique microflora with interesting traits (Cavicchioli, 2015). One of the biggest hurdles in such places is the energy equity generated by various essential drivers such as sunlight cycles, water, and nutrients, oceanic limnology influencing the biological activities. Bioprospection of such unique ecological niches has revealed a plethora of microorganisms (psychrophiles/psychrotolerants) with intriguing survival methods (Prasad et al., 2014). Both psychrophiles (cold-loving) and psychrotolerants (cold-tolerant) possess a major difference in the range of their growth temperatures; former has the ideal growth around 15°C reaching to the maximum temperature around 20°C and a minimum temperature of 0°C or below while latter is adapted to thermal fluctuations as well (Margesin and Feller, 2010; Dhakar and Pandey, 2020; Kanekar and Kanekar, 2022). A few examples of psychrophilic *Pseudomonas* spp., reported are *P. helmanticensis*, *P. antarctica* sp. nov., *P. meridian* sp. nov., *P. proteolytica* sp. nov., *Pseudomonas* sp. LSK25 (Reddy et al., 2004; Salwoom et al., 2019; Kumar et al., 2020) and psychrotolerant *Pseudomonas* spp., are *P. palleroniana* (GBPI_508), *P. syringae*, *Pseudomonas* sp. NB-1, *Pseudomonas* sp. MPC6 (Kumar et al., 2002; Selvakumar et al., 2009; Orellana-Saez et al., 2019; Jain et al., 2020; Cui et al., 2022; Thathola et al., 2022).

Microorganisms, as the simplest yet most unique life forms, extend tremendous applications in various sectors. They are the most numerous and diversified organisms present in almost all the extreme conditions on earth. The implication that microbes are necessary for life has attracted attention in microbial diversity research as they perform a variety of functions, critical to the biosphere’s survival. Prominent examples are the biogeochemical cycles and environmental biotransformation involving processes such as nutrient augmentation, recycling, and supplementation which are critical to environmental sustainability (Deming, 2002; Yadav et al., 2015a).

The metabolism of psychrophilic microorganisms is influenced by abiotic factors such as temperature, pH, salinity, organic content, and inorganic nutrients. These factors are also responsible for the composition of various groups of microorganisms. Based on a comprehensive literature survey, major inhabiting psychrophilic groups comprise of *Proteobacteria*, *Bacteroidetes*, *Cyanobacteria*, *Actinobacteria*, *Firmicutes* and *Chlamydiae* (Annapure and Pratisha, 2022). Psychrophiles belonging to *Bacillus* such as *B. megaterium, B. amyloliquefaciens* (Verma et al., 2015a)*, Bacillus*-derived genera (BBDG), *Pseudomonas gessardii* (Kaur et al., 2021) have been observed as pigmented organisms that synthesize exopolysaccharides, antibiotics, antifreezing compounds, antagonistic amalgams, and cold active enzymes (Yadav et al., 2018; Silva et al., 2021). The hypothesis stating whether a microbiome trapped in a cryosphere for an extended period of time results in effective growth stimulation has been proven correct. In this context, numerous examples support the fact that species of *Pseudomonas* inhabit a diverse variety of niches due to physiological flexibility. They can adapt to high and low temperatures, oxygen, moisture and nutrients (Sherpa et al., 2019; Snopková et al., 2020; Cui et al., 2022; Suyal et al., 2022). *Pseudomonas* can use a wide range of organic substances as carbon and energy sources. Some species are capable of utilizing a variety of toxic compounds via production of a myriad of secondary metabolites and are being recognized in agriculture, medicine, and other industries (Orellana-Saez et al., 2019; Yadav et al., 2019; Craig et al., 2021; Thathola et al., 2021, 2022).

The microbial communities have been the focus of chosen scenario, not just in respect of biotechnological prospects, but also in terms of understanding how primitive counterparts of biomolecules were used in early Earth habitats. The field of bioprospecting research is quickly expanding using various approaches, technologies, and methodologies as well as the scientific rationales and hypotheses. The concerned areas in the current review majorly include the Indian Himalayan region (IHR) – that forms a large part of Himalayan global biodiversity hotspot along with reports from the neighbor countries; Tropical Andes- a huge region in South America stretching along Andes mountains from Southwestern Venezuela to Northern Chile and Argentina, regarded as the origin of agriculture in America; and Antarctica the fifth largest southernmost continent consisting of 95% of the ice coverage. The critical factor differentiating Antarctica from the rest is the detrimental solar radiations and variable photoperiod (Chettri et al., 2008; Pandey and Yarzábal, 2019; Sharma et al., 2019; Rizzo et al., 2021; Yarzábal, 2021).

## Factors regulating microbial diversity in cold environments

Most studies utilize temperature dependency criteria to designate psychrophiles, however soil nutrition also plays a significant role in growth determination. As a result, the available substrate regulates metabolism and changes the enzymatic rate. Owing to the mineral absorption tactics, certain strains of *Pseudomonas* intensively invade roots and begin rapid root colonization and develop host-microorganism interaction and a tactical defensive pathway to regulate neighboring microorganisms. These properties allow *Pseudomonas* to inhabit most of the nutrient deficient locations. *Pseudomonas* tends to degrade a wide range of compatible solutes such as heterosides, aminoacids and sugars (Craig et al., 2021) and organic compounds adopting various metabolic pathways such as Entner-Doudoroff pathway, β-ketoadipate pathway (Cabrera et al., 2022) etc. The psychrophiles have been considered to possess the ability of tolerating far higher temperature ranges than the real ambient temperature from which they were isolated, when cultivated *in-vivo* (Cavicchioli, 2016); for example, *P. psychrophila* MTCC12324 (Abraham et al., 2020) and *P. frederiksbergensis* (Cui et al., 2022) etc., were observed to grow between 4–37°C, *Pseudomonas* sp. 30–3 with a survival range of 0–35°C retrieved from oil-affected soil, Wright Valley, Antarctica (Panicker et al., 2002); *P. lundensis,* isolated from Antarctic meltwater pond was also reported to survive at 0°C (Ravi et al., 2022). Similarly, pH is another important factor regulating the survival and growth of microorganisms in extreme climatic conditions. Microorganisms, isolated from extreme temperature environments revealed the ability to grow at wide pH range, extreme acidic to extreme alkaline (Dhakar and Pandey, 2016). Some of the examples include *P. stutzeri*, *P. frederiksbergensis, P. simiae*, *P. brenneri*, *P. azotoformans* from Kongsfjorden and Ny-Alesund, Svalbard, Arctic (Srinivas et al., 2009); and *P. corrugata, P.* PGERs17, *P. putida* B0, *P. chlororaphis* GBPI_507 (MCC2693) from different regions in Indian Himalaya (Pandey and Palni, 1998; Pandey et al., 2006; Mishra et al., 2008; Jain and Pandey, 2016). It was connected to the concept that the genome is a probable repository of hidden variants. While extremophilic microorganisms have the potential to adapt to the changing environmental conditions, this is most likely accomplished through the expression/regulation of certain genes originally existing in the genome (Dhakar and Pandey, 2016).

Elevation is another important factor affecting the growth of microorganisms in a given set of climate. In the Colorado Rocky Mountains, Bryant et al. (2008) reported a linear decline in soil microbiome diversity as elevation climbed. Bhattacharya et al. (2022a) strongly suggested the influence of edaphic factors on microbial diversity using meta-barcoding based analyses. The report indicated an almost similar diversity at high as well as low elevation; however at mid-elevational gradient the fluctuation was recorded due to the soil moisture, organic carbon, and total nitrogen content. The authors also reported no significant microbial diversity variation in annual and seasonal patterns. The importance of edaphic factors on microbial community composition and their metabolism was studied in Gangotri glacier’s post-deglaciation patterns during different time span (Bhattacharya et al., 2022b). Another notable finding from Indian Himalayan region (IHR) research is the influence of mountain slope orientation on several bioactivities displayed by plant growth promoting microorganisms colonizing these habitats (Selvakumar et al., 2009). Nottingham et al. (2018) also noted the coordination pattern of elevation, temperature, and species (plants and microbial) diversity in Andes (Peru). They considered temperature as the major diversity determinant with less influence of edaphic factors (soil pH). They also suggested that the enzymes responsible for organic matter degradation were accompanied in microbial transition patterns taking place along the elevation. As a result, in mountain ecosystems, climate conditions work together to favour the colonization and dominance of specific microbial populations. Dominance of cold adapted microbial communities has been documented to colonize tree species of IHR namely *Abies, Rhododendron* and *Betula* in alpine and glacier environments along an altitudinal gradient (Pandey and Palni, 2007).

## *Pseudomonas*: A diverse and important genus

*Pseudomonas* is ubiquitous, inhabiting the atmosphere, terrain, and hydrosphere while surviving a broad range of topographical characteristics. While Parte (2018) refers to the List of Prokaryotic Names withstanding in Nomenclature with 122 validly published species of *Pseudomonas*, Gomila et al. (2015) describes the major species groupings as *P. aeruginosa*, *P. syringae, P. chlororaphis*, *P. putida*, and *P. fluorescens*.

Low temperature environments have been ideal sites for colonization of cold adapted *Pseudomonas* species, both psychrophilic and psychrotolerant. Novel psychrophilic Pseudomonads namely *P. antarctica* sp. nov., *P. prosekii, P. meridiana* sp. nov., *P. proteolytica* sp. nov., *Pseudomonas* sp. ID1 have been isolated from low temperature environments of Antartica (Reddy et al., 2004; Carrión et al., 2015; Snopková et al., 2020). Diversity of the genus *Pseudomonas* including *P. azotoformans, P. poae, P. fluorescens, P. reactants, P. hibiscicola* and *P. synxantha* has been reported from Kanchengayao glacier, North Sikkim Himalaya, India, as one of the most dominant genera in the glacier ice samples (Sherpa et al., 2019). Other psychrophilic species namely *Pseudomonas* sp. BGI-2 from Batura glacier, Pakistan (Ali et al., 2020), and *P. frederiksbergensis* ERDD5:01 isolated from the East Rathong glacier in the Sikkim Himalaya have been reported for their resistance to high altitude stress conditions such as prevalent freeze–thaw patterns and UV radiation (Kumar et al., 2019). *Pseudomonas* species namely *P. azotoformans, P. corrugata, P. granadensis, P. paralactis, P. proteolytica, P. palleroniana, P. helmanticensis* and *P. putida* have been isolated from various locations including cold deserts and glacial sites in Indian Himalaya (Pandey et al., 2019; Kumar et al., 2020). *P. psychrophila* MTCC12324, a cold adapted strain has been reported for its specificities for inhabiting the Arctic region (Abraham et al., 2020).

Cold adapted *Pseudomonas* species, with plant growth promotion (PGP) activities, have been identified from Himalayan and Andean mountain environments (Balcázar et al., 2015; Pandey and Yarzábal, 2019). Pseudomonads have been identified as the most physiologically varied bacterial immigrants to alpine soils, with the ability to thrive across a wide temperature range while resisting a variety of biotic and abiotic challenges. As a consequence, it exhibits its use as a bioinoculant in overcoming hill agricultural challenges. The low temperature environment will have an influence on cellular structure due to osmotic imbalance, water content, membrane fluidity, enzyme kinetics, and diffusion rate. Some traits must be different in order to maintain the critical metabolism (De Meeyer et al., 2014).

## Adaptation strategies – A facet of versatility

**Figure 1:**
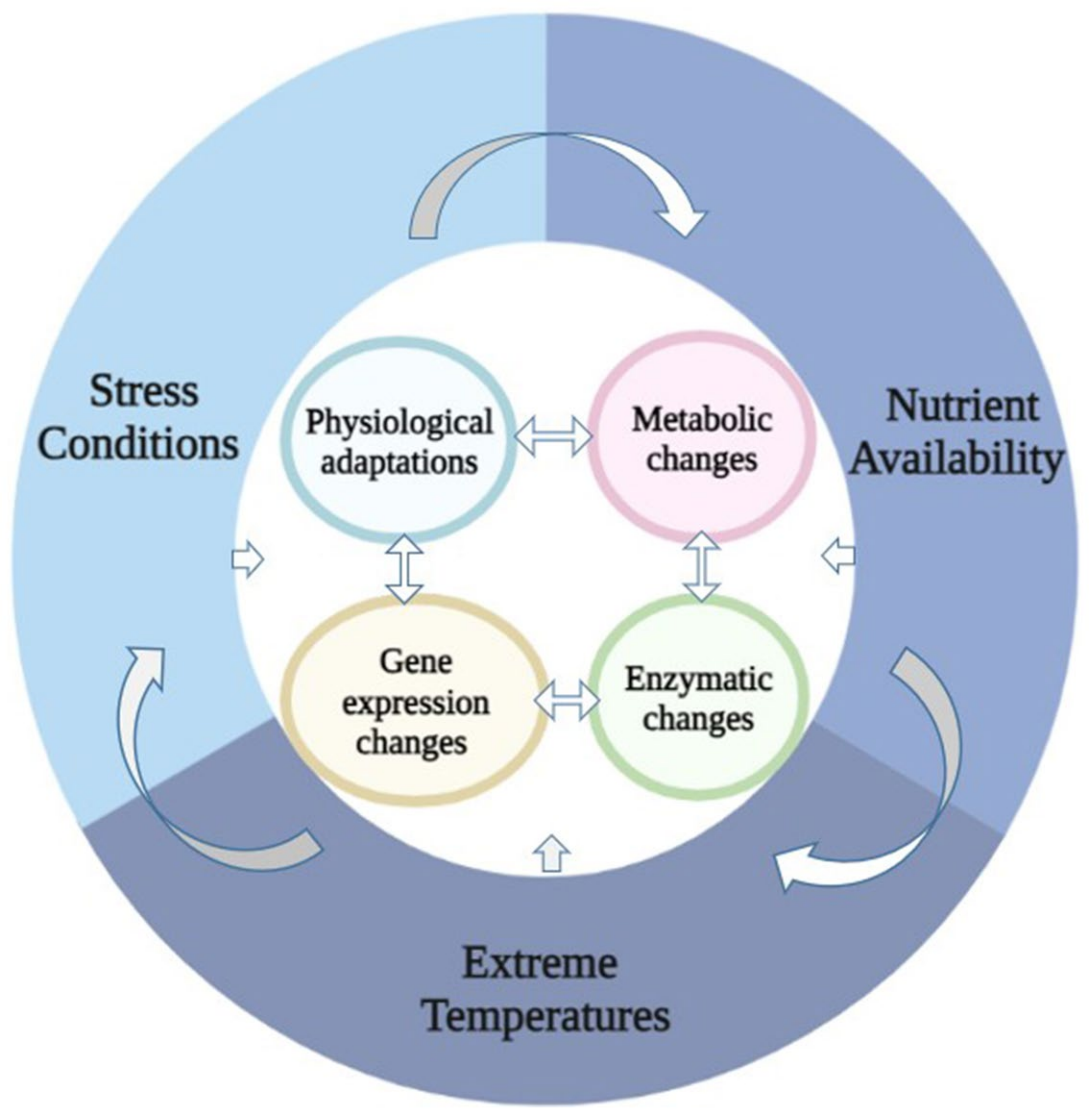
Impact of environmental factors and their interaction to cell functioning.

Diverse topographical, ecological, and biological factors form the basis of physiological and molecular acclimatization (Figure 1). *Pseudomonas* are known to acclimatize to low temperature via physiological changes (Table 1) like membrane integrity, production of anti-freeze proteins, carotenoid pigments, the release of cryoprotectants, basic metabolic adjustment such as suppression of glycolysis and TCA cycle during cold and the acquisition of energy generation by secondary or intermediate molecules via alternate routes to circumvent the whole system emerge as significant aspects for energy production (Tribelli et al., 2015; Abraham et al., 2020).

**Table 1:** Temperature tolerance and survival strategies of some *Pseudomonas* species.

S. No.	Species name	Habitat	Temperature	Survival strategies	References
1.	*P. proteolytica*, *P. brenneri*, *P. psychrophila* E3	Andean region	4–20°C	Abundant pigment and exopolymers production	Ball et al. (2014)
2.	*P. extremaustralis*	Antarctica	8–30°C	PHB production and accumulation, alternative energy production and ethanol oxidation	Tribelli et al. (2015)
3.	*P. chlororaphis* MCC2693	Indian Himalayan region	4–25°C	Production of phenazine and siderophores	Jain and Pandey (2016)
4.	*P. extremaustralis*	Antarctica	8–30°C	LPS modifications biofilm and elasticity of cell envelope structures	Benforte et al. (2018)
5.	*P. helmanticensis*, *P. putida*	Antarctica	4–30°C	Production of cryoprotective polyamines	Tribelli and López (2018)
6.	*Pseudomonas* sp. LSK25	Antarctica	0–30°C	Cold -adaptive lipase production	Salwoom et al. (2019)
7.	*Pseudomonas* BGI-2	Batura glacier, Pakistan	4–35°C	Production of cryoprotective membrane and agents	Ali et al. (2020)
8.	*P. helmanticensis*	Indian Himalayan region	2–20°C	Expression of novel proteins, ROS neutralizing enzymes, molecular chaperones, and unsaturated fatty acid biosynthesis	Kumar et al. (2020)

The capacity of the cell to control the fluidity of the membrane is one key tactic that is essential to the cell’s survival at low temperatures. A robust hydrophobic lipid membrane is required for all biological membranes. *Pseudomonas* genera generally modify membrane fluidity by changing the lipid polar head, charge, constituents, size and location, length and arrangement of hydrophobic fatty acid chain, peptidoglycan biosynthesis, membrane’s protein composition, amino acid stocking, kind of carotenoids generated, and ratio of cis to trans fatty acids (De Meeyer et al., 2014). Certain reports suggested that *Pseudomonas* may have a two-part signal transduction system that consists of a membrane confined sensor and a dissolvable cytoplasmic response regulator that is involved in the perception and transmission of low temperature signals (Chintalapati et al., 2004; De Meeyer et al., 2014; Raymond-Bouchard et al., 2018; Weinstein et al., 2019; Abraham et al., 2020). *P. syringae, P. aeruginosa,* and *P. extremaustralis* are a few examples reported from Antarctic, Arctic, and IHR, known to exhibit similar phenomenon (Kumar et al., 2002; Benforte et al., 2018). The breakdown of amino acids behaves as one of the pathways that resulted in the production of the cytoprotective polyamines as observed in *P. helmanticensis* (L-arginine degradation leads to the formation of spermidine and putrescine) and *P. putida* (Tribelli and López, 2018; Kumar et al., 2020).

According to proteomic research, transport proteins discovered in the membrane have been reported to compensate for poor diffusion rates by increasing the absorption of suitable solutes, nutrients, and peptides for peptidoglycan formation (Cacace et al., 2010). Chevalier et al. (2017) supported the fact and observed similar outcomes in *P. aeruginosa*; they express specific channel proteins for nutrient uptake. Exopolymeric compounds are known to be produced in large quantities by bacteria living in cold aquatic environments (e.g., Antarctic and Arctic Sea ice). These are hypothesized to help cells attach to wet surfaces and create the extracellular matrix structure of biofilms (owing to the development of a large quantity of alginate; main ingredient of glycocalyx), which absorbs nutrients, protects the cell from adverse ecological parameters, and facilitates metabolic interactions (Palleroni, 2010; Yadav et al., 2015c; Ciofu and Tolker-Nielsen, 2019; Abraham et al., 2020). *P. mandelii*, a novel strain isolated from Antarctic environment, has been reported for overproduction of alginate in comparison to other species like *P. aeruginosa* and *P. fluorescens*. According to this study, the expression levels of the alginate operon were recorded highest at 4°C (Vasquez-Ponce et al., 2017). The high concentration of EPS lowers water’s freezing point and ice nucleation temperature. They must also account for increased pore density and disorganized ice crystals, which reduce permeability and salt retention (De Meeyer et al., 2014).

The evolution of cryoprotective carbohydrates is one of the significant features of pschryophilic microorganisms. Numerous examples belonging to *Pseudomonas* genera, such as *Pseudomonas* sp. ID1 and *Pseudomonas* sp. BGI-2 have been reported to form such exopolysacchrides conferring significant cold adaptation (Carrión et al., 2015; Ali et al., 2020). Furthermore, the culture media, growth substrates and environmental factors can be optimized for enhanced yield (Ali et al., 2020; Nair et al., 2021).

Lowering the temperature has an inverse effect on enzyme kinetics, with a 10°C drop, the enzyme activity decreases by two fold. Psychrophilic enzymes (phospholipases, proteases) maximise low temperature activity by destabilizing the structures containing the active site as well as the whole molecule. This attributes to a decrease in the strength of intramolecular connections (subunit, electrostatic interactions, disulfide linkages) (Rapuano and Graziano, 2022) and the exclusion of stability factors, resulting in better active site dynamics (Feller, 2013). Other factors responsible for the flexibility of such enzymes include enhanced surface, core hydrophobicity and the arrangement of aminoacids. Some of the latest reports strongly support that the presence and orientation of specific amino acids within the structure of enzyme affects the stability and activity, for example, the catalytic triads Ser, His and Asp in lipase encoding genes found in *P. marinensis* were observed to be necessary for preserving enzyme functions (Guo et al., 2021). Öten et al. (2022) observed a boost in the adaptation of enzymes to cold conditions by proline (Pro) and glycine (Gly) residues modifying conformational changes in the three-dimensional (3-D) structure of proteins. The study demonstrated the mechanism of Gly and Pro in the cold-adaptive model, the inclusion of Gly residue enhances the flexibility of the peptide backbone, allowing conformational changes (Raymond-Bouchard et al., 2018). Gly is entirely made of hydrogen which provides the property of being achiral, and the inability to participate in intramolecular interactions promoting the destabilization of helix structures. On the other hand, the bulky N-CH_2_ groups of Pro, successive residues have limited conformations, affecting helix formation. If Pro appears on the first turn of the helix and aligns closely to the backbone shape, it causes a significant influence on protein stabilization (Öten et al., 2022). Lipase, protease, amylase, cellulose, urease and beta-glucosidase are some of the reported enzymes from *Pseudomonas* sp. (Choo et al., 1998; Salwan et al., 2010; Jain et al., 2017; Salwoom et al., 2019; Salwan et al., 2020; Rubiano-Labrador et al., 2022). The stability of secondary structures in cold temperature were studied and determined by Gianese et al. (2001). The authors suggested the following substitutions of charged residues such as Val (omission in beta strands), Ala (presence in coiled region), and Glu, Lys and Arg (at the alpha helix and coil regions) to favor psychrophilic adaptation. Extracellular metalloprotease extracted from *Pseudomonas* sp. (Yang et al., 2010) and protease (Martínez-Rosales and Castro-Sowinski, 2011) correspond to diverse industrial applications.

The synthesis of polyhydroxyalkanoates (PHAs), such as, polyhydroxybutyrate (PHBs) was discovered to be critical for microbial growth, tolerance and survival to a number of environmental stressors while possessing environmental significance. PHB accumulation maintains cellular redox homeostatis, cope oxidative stress, boosted motility and survival in *P. extremaustralis*, indicating that the capacity to accumulate PHB might be an adaptive benefit for colonizing new biological niches in such circumstances (Tribelli and López, 2011).

Cold temperatures, as encountered in higher altitudes and polar region, disrupt nucleic acid structure hampering protein structures and basic molecular processes. In order to deal with these, certain cold adapted microorganisms produce specialized proteins known as ice-nucleating proteins (INPs) and antifreeze proteins (AFPs). The role of each differs; AFPs prevent the production of large ice crystals within the cell which can damage organelles further resulting in cell death whereas INPs are linked with the cell membrane, they induce extracellular ice generation preventing the freezing of cytosolic water (Białkowska et al., 2020; Craig et al., 2021). *P. putida* GR12-2 was reported to release an AFP (AfpA protein) that sustain life at subzero temperature and exhibit both ice nucleation as well as antifreeze abilities (Muryoi et al., 2004). The INPs from *P. syringae* and *P. borealis* were taken as model to study their binding mechanisms (Hudait et al., 2018). These species play a pivotal role in food preservation, cryopreservation, material technology etc. *P. helmanticensis* (Kumar et al., 2020), *P. psychrophila* MTCC12324 (Abraham et al., 2020), *P. prosekii* (Snopková et al., 2020) demonstrated the potential of genes encoding for cold shock proteins serving as nucleic acid chaperons and prevents the misfolding of mRNA to withstand freezing patterns.

Another ecologically as well as biotechnologically important adaptation possessed by psychrophiles is pigment formation in order to confront ultraviolet and oxidative stress conditions. Pigments are one of the characteristic properties of such cold adapted organisms that are being researched for numerous industrial uses. They are largely produced inside the cytoplasm as a response agent to harsh ecological circumstances, serving a variety of ecological as well as physiological purposes including membrane fluidity regulation, cell differentiation, and photo-oxidative injuries (Sajjad et al., 2020). A few pigments regulate iron concentration and metabolism (Dhakar and Pandey, 2020). *P. aeruginosa, P. chloraphis, P. reptilivora, P. stutzeri, P. corrugata, P. mendocina* and *P. fluorescens* are some of the most widely known species to release blue-green pyocyanin or yellow-green fluorescein (pyoveridine), orange chlororaphin, reddish brown pyorubin, and brownish black pyomelanin pigments (Dhakar and Pandey, 2020). The pigments possess a variety of industrial applications and have a good market potential (Venil et al., 2020), thus has been an interesting objective for researchers.

On account of genetic transformation, certain modifications include chaperone formation responsible for efficient protein folding and nucleic acid machinery in order to generate a stable secondary structure at low temperature. Phosphorus mobilization is aided by the expression of mineral solubilizing genes such as pyrroloquinoline quinine (pqq) and glucose dehydrogenase (gcd) (Rawat et al., 2021). The specialized porin genes (OrpF, OprB, OprH, OprP etc.) help regulate adherence, relay signals, nutrient propagation, and anatomical functions reported in *P. aeruginosa* (Chevalier et al., 2017).

**Figure 2:**
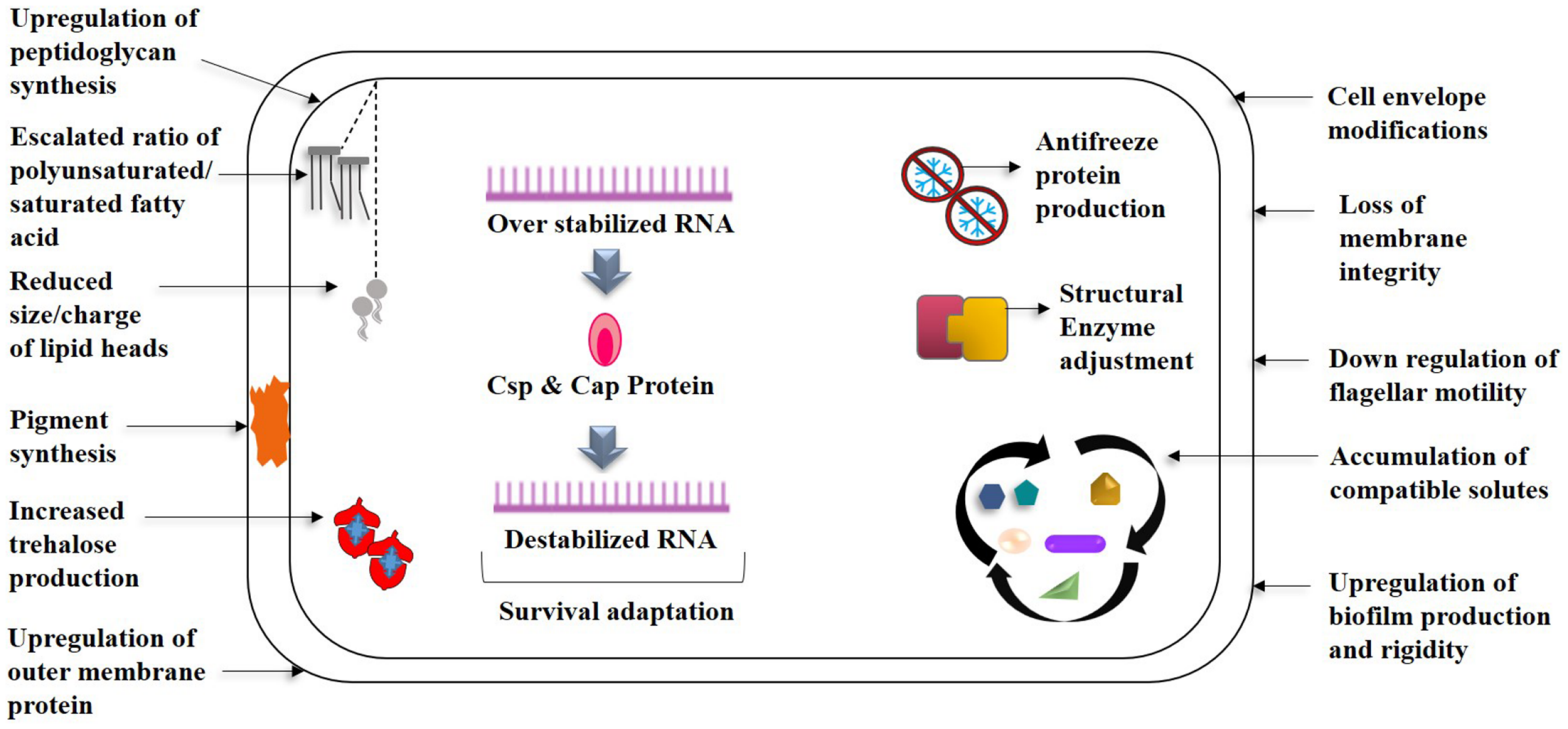
Cellular mechanisms of *Pseudomonas* spp. towards the survival strategies in low temperature- An overview.

**Figure 3:**
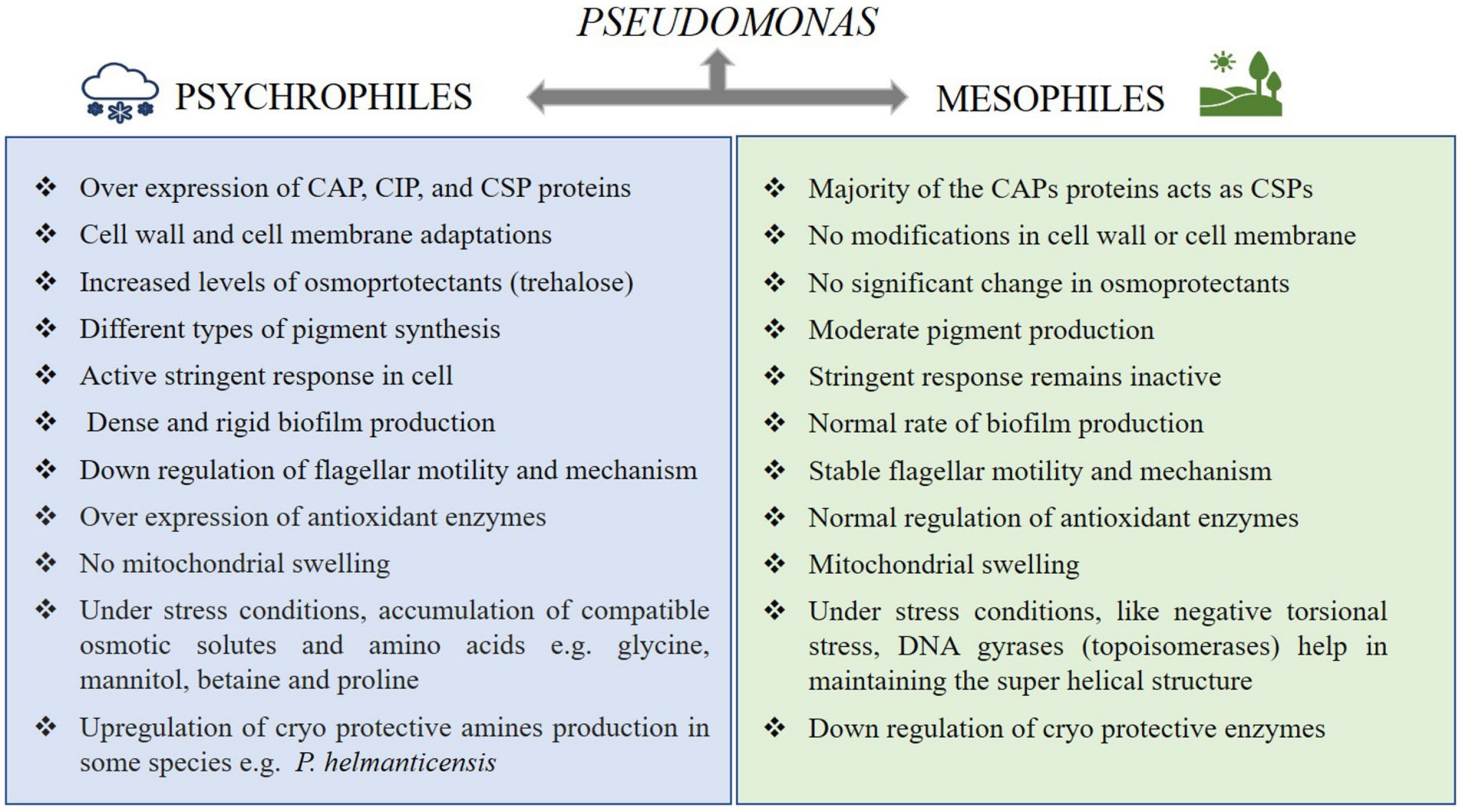
Key differences in psychrophilic and mesophilic members of the genus *Pseudomonas*.

Multiples of cold associated genes encoding cold active chaperons, for example, *HscA/HscB, CspD, GroES, GroEL, HtpG* etc., carbon storage/starvation, overall stress response (*Ctc*, *UspA*, *LexA* etc.), oxidative stress, membrane/cell wall modification, osmotic pressure, and DNA repair mechanisms (recombinase RecA and RecQ) aid in the resistance of *P. frederiksbergensis* ERDD5:01 and *P. prosekii* to extreme cold and radiations correlating with bacterial physiological observations (Kumar et al., 2019; Snopková et al., 2020). Genes encoding cold shock domain such as capB was suggested by Panicker et al. (2002) as a cold survival promoter which was later proven due to its presence in all the *Pseudomonas* spp., isolated from Antarctica (Panicker et al., 2010). UV-radiation has a significant impact on higher altitude and cryospheric areas, and *Pseudomonas* has several photoreactivation genes, such as cyclobutane pyrimidine dimers (CPDs), to assist against radiation stress (Kumar et al., 2019). Plasmid (for example, *P. fluorescence – E. coli* shuttle plasmid, *P. chlororaphis* PA23) genes usually contribute in organisms’ quick adaptation processes by enhancing the phenotypic flexibility of host strains via horizontal gene transfer, hence assisting them in cold tolerance (Dziewit and Bartosik, 2014; Ghergab et al., 2021; Wu et al., 2021). Figure 2 depicts a diagrammatic description of the aforementioned adaptations. The distinguishing characteristics of psychrophilic and mesophilic *Pseudomonas* are summarized in Figure 3.

## Potential applications of *Pseudomonas*

Aboveground and belowground microorganisms have been shown to have substantial ability in dealing with diverse ecological stressors and nutritional deficit. Microorganisms and plants interact through associative, endophytic, or symbiotic processes with varying degrees of proximity to the roots and rhizosphere (Kaur et al., 2021). Agricultural sustainability is supported through microbial interactions. Among cold adapted microorganisms, dominance and biotechnological applications of *Pseudomonas* species have been investigated for their contribution mainly in agriculture, and industries (Table 2).

**Table 2:** Ecological and biotechnological applications of different species of *Pseudomonas.*

S. No.	Name of the species	Applications	References
1.	*P. putida NRRL B-30041	Biocontrol- against repellent disease in tree fruits	Mazolla (1999)
2.	*P. proteolytica* sp.nov CMS 64^T^	Biocontrol property	Reddy et al. (2004)
3.	*Pseudomonas* sp. CIBEA9	Cold-active biofertilizer	Yarzábal et al. (2018)
4.	*P. putida* (B0)	Plant growth promoting (PGP) traits-Solubilization of tricalcium phosphate	Pandey et al. (2006)
5.	*P. corrugata*	Bacterial inoculant, increase in maize yield (PGP)	Kumar et al. (2007)
6.	*Pseudomonas* sp. gb3 (MTCC 9476)	Propagation and conservation	Pandey et al. (2009)
7.	*P. frederiksbergensis*, *P. peli*, *P. putida, P. reactans*, *P. stutzeri*	Bioinoculants- Plant growth promotion at low temperature	Yadav et al. (2015a,b)
8.	*P. palleroniana* R4363	Bioinoculant- increase in potato tuber number and yield	Velivelli et al. (2015)
9.	*P. fragi*, *P. fredericksbergensis*, *P. brenneri*, *P. fluorecens*, *P. psychrophila*	Cold- active biofertilizer potential	Balcázar et al. (2015)
10.	*P. chlororaphis* GBPI_507 (MCC2693)	Plant growth promotion and biocontrol properties	Jain and Pandey (2016)
11.	*Pseudomonas* sp.CIBEA9	Improved growth of wheat seedlings	Yarzábal et al. (2018)
12.	*Pseudomonas* spp. PGV045, PGV094, PGV233, PGV274	Improved growth and disease control in wheat	Rondón et al. (2019)
13.	*P. corrugata*, *P. fragi*, *P. putida*, *P. vancouverensis*	Growth promotion in agricultural, forest and plantation species	Pandey and Yarzábal (2019) and reference within
14.	*Pseudomonas* sp. BGI-2	Production of cryoprotective membrane and agents	Ali et al. (2020)
15.	*P. nitroreducens* TX1	1 Bioremediation of nonionic surfactants and estrogen-like alkylphenols	Huang et al. (2014)
16.	*P. nitroreducens ATCC PTA-6168, (BCRC910228)	2 Bioremediation of organic polymers	Huang et al. (2004)
17.	*P. stutzeri LH4:15	3 Bioremediation- enhanced oil release through microbial consortium	Keeler et al. (2008)
18.	*Pseudomonas* sp. MPC6	4 Bioremediation of arsenic, cadmium, alkanes, chloro- and nitro-aromatics compounds at low temperature	Orellana-Saez et al. (2019)
19.	*Pseudomonas* sp. ef1	Bioremediation of silver nitrate contaminants	John et al. (2020)
20.	*Pseudomonas* sp. (GBPI_Hb5), (MCC 3295)	Caffeine degradation	Thathola et al. (2021)
21.	*P. palleroniana* GBPI_508	Biodegradation of bisphenol A	Thathola et al. (2022)
22.	*P. antarctica* spp. ERGC2:04, ERGC3:01, ERGC3:05, ERCE:11	Poly adaptative potential in cosmetic, detergent, and pharmaceutical industries	Mukhia et al. (2022)
23.	*P. mandellii* IHB 5670	Use in Detergent industry	Salwan et al. (2020)
24.	*P. geniculata* ATCC 19374	Livestock waste water treatment	Liu et al. (2021)
25.	*P. burkholderia, *P. aeruginosa	Lipase production	Friedrich and Stürmer (2003)
26.	*Pseudomonas* sp. LSK25	Lipase production	Salwoom et al. (2019)
27.	*P. marinensis gcc21*	Lipase production	Guo et al. (2021)
28.	*P. fluorescens Fe3	Food production (vanillin)	Narbad et al. (2003)
29.	*P. putida ATCC 53552*	Use in Textile industry	Mizusawa et al. (2001)
30.	* *P. cichorii*	Use in Textile industry	Imamura et al. (2002)
31.	*P. putida NClMB 8,859	Use in pharmaceuticals- resolution in cis 1,2- indane diols	Allen et al. (2000)
32.	* *P. syringae*	Use in pharmaceuticals- pseudomonic acid preparation	Szell et al. (2001)
33.	*Pseudomonas sp. 19/26	Use in pharmaceuticals -pseudomycin production	Hilton et al. (2009)
34.	*P. vesicularis, *P. maltophilia	Cosmetics	Martin et al. (2002)

* Patented strains.

## Plant growth promotion

Plant growth promotion (PGP) responsibilities can be mediated by indirect or direct or both kinds of mechanisms. The former is closely related to biological management of plant diseases, and relies on the manufacture of antibiotics, lysozymes, siderophores, and biocidal metabolites. Some microbial secondary metabolites, such as the generation of phytohormones, release of bioavailable nitrogen to the host, and phosphate solubilization, also contribute to support the plant’s defense responses (Pandey and Yarzábal, 2019). Plant growth promoting properties mainly consisted of production of phytohormones, IAA, HCN, siderophore, ACC deaminase activity, mineralization of K, P, Zn, and ethylene biosynthesis inhibition (Balcázar et al., 2015; Ortiz-Ojeda et al., 2017; Kaur et al., 2021). Psychrophiles also produce phytohormones, including gibberellins, cytokinins, and auxins; the most significant modulators of physiological and molecular responses.

Several research groups have expressed interest in developing cold active biofertilizers and biopesticides to promote sustainable agriculture in mountainous regions (Pandey and Yarzábal, 2019; Yadav et al., 2019; Torracchi et al., 2020). Numerous field trials, directed at determining the impact of PGP microorganisms on vegetation, have declined due to exogenous microorganism’s low survival rates when introduced to soils from another location due to the fact that a 10°C temperature decrease may result in around two-fold reduction in metabolism (Feller and Gerday, 2003). Thus, it was proposed that microorganisms intended for use as bioinoculants be prospected and selected from their own inhabiting microbial communities (Velázquez et al., 2016).

The development of microbial formulations for boosting agricultural productivity in cold conditions was considered to be reliant on two crucial factors: the microorganisms’ environmental resilience and rhizosphere proficiency. Psychrophiles are known to have significant ecological traits in addition to acquired survival skills. A field research done at various elevations in the Mamlay watershed of the Sikkim Himalaya (Pandey et al., 1998) demonstrated the relevance of local microorganisms and the necessity for cold adapted PGP microorganisms to be employed in mountain ecosystems. The study also demonstrated the feasibility of cold adapted *Pseudomonas* (*P. corrugata*) for development as bioinoculants for maize grown in higher altitudes (Kumar et al., 2007). Later, PGP features of a number of *Pseudomonas* species, namely *P. corrugata*, *P. fragi*, *P. putida*, *P. vancouverensis*, and *P. libanensis* EU-LWNA-33, isolated from various mountain locations were documented (Negi et al., 2011; Kour et al., 2020). A polyextremophilic *P. chlororaphis* (MCC2693) strain from the Indian Himalaya, that produced phenazine-1-carboxylic acid in culture conditions, was shown to promote plant growth in bioassays (Jain and Pandey, 2016).

*Pseudomonas* strains isolated from the rhizosphere of potato cultivated at various elevations in Peru and Bolivia’s Central Andean Highlands were shown to have a variety of PGP activities (Ghyselinck et al., 2013). Psychrotolerant PGP bacteria were isolated from the rhizosphere of a tuber crop, *Lepidium meyenii* Walp. (maca), of the Andean region (Ortiz-Ojeda et al., 2017). The diversity of rhizosphere bacteria colonizing tubers, one of the most important staple crops in the Andean highlands, has been reported for the dominance of *Pseudomonas* and *Bacillus* strains with PGP activities (Chica et al., 2019). ILQ215 (*P. silesiensis*) and JUQ307 (*P. plecoglossicida*), isolated from *Chenopodium quinoa* rhizosphere with considerably benefitting plant growth impacts and the potential for producing bioinoculants, have been investigated from the Peruvian Andean Plateau region (Chumpitaz-Segovia et al., 2020).

Investigation on the genetic diversity and PGP activities of wheat rhizosphere related bacteria from the northern hills of India was undertaken and reported the dominance of *Pseudomonas* (Verma et al., 2015b). The analysis revealed the importance of cold adapted microorganisms in producing bioformulations for use in winter crops cultivated in high altitude prevailed by low temperature climatic conditions (Verma et al., 2015a). PGP positive *Pseudomonas* isolates (next to *Bacillus*) have been reported to predominate the frigid desert of Leh Ladakh in Indian Himalaya (Yadav et al., 2015a).

Several *Pseudomonas* species are reported to have PGP activities such as mineral phosphate solubilization (da Silva et al., 2021) and the generation of chemicals that contributes to plant development or suppress plant pathogens; indole acetic acid (IAA), 1-aminocyclopropane-1-carboxylate (ACC) deaminase, ammonia, and HCN. Furthermore, these cold adapted *Pseudomonas* species have been shown to relieve plant stress induced by several environmental conditions (Mishra et al., 2011; Ma et al., 2017). Many biostimulants, regardless of the nutrients they contain, enhance nutrition. Subramanian et al. (2015) investigated the biostimulant potential of cold adapted endophytic *P. vancouverensis* OB155-gfp and *P. frederiksbergensis* OS261-gfp in *Solanum lycopersicum* Mill. da Silva et al. (2021) isolated *Pseudomonas* sp. 11. LB15 and *Pseudomonas* sp. 1.LB34 from Antarctic lichen that was capable of phosphate solubilization and also released tartaric and fumaric acids during the process.

*Pseudomonas* genus has been successfully reported for antagonistic behaviour against diverse phytopathogens. For instance, *P. corrugata* isolated from Sikkim (maize field) with an ability of growing at 4°C was tested to protect *Cedrus* plants from cutworms and *Fusarium* wilt at nursery stage (Bisht et al., 2003). *P. putida* B0, an isolate from IHR, was found to inhibit the growth of *Phytophthora parasitica* (Pandey et al., 2006). PGP activities of *Pseudomonas* spp. obtained from glacier samples in Venezuela’s tropical Andes were investigated by Balcázar et al. (2015). While some of the bacterial isolates were psychrophilic, others grew at a wide range of temperature. *Pseudomonas* spp. collected from Greenwich Island soil, Antarctica thrived well at temperatures between 4 to 30°C and screened positive for PGP characteristics. Furthermore, the bacteria suppressed phytopathogens such as *Fusarium oxysporum, Phytophthora infestans* and *Pythium ultimum* and promoted germination of wheat seeds. The researchers concluded that the selected Antarctic *Pseudomonas* spp. can be developed as cold active biofertilizers (Yarzábal et al., 2018). A few *Pseudomonas* spp., retrieved from Venezuelan glaciers, have been demonstrated for their antagonism behaviour against *P. ultimum* in order to induce growth in *Triticum* (Rondón et al., 2019).

In the coming years, agriculture is likely to confront the combined task of feeding a growing global population while also reducing the environmental effect of cropping systems. *Pseudomonas* retrieved from the cryosphere has long been employed as potent inoculants for carrier-based formulations; they were thoroughly investigated for their plant growth boosting properties at low temperatures. Trivedi et al. (2005) demonstrated this point with a successful formulation of *P. corrugata* covered with alginate beads. Moreover, it has been shown that the creation of biostimulants acquires a potent mechanism that boosts plant development, improves nutrient use efficiency, and generates novel mechanisms for plant nutrient acquisition and abiotic stress tolerance (Du Jardin, 2015), resulting in minimizing the need of chemical fertilizers. Advancement in research with respect to development of carrier based formulations, applicable to cold environments retaining the desirable PGP traits, will be necessary for large scale production and commercialization of this technology.

## Bioremediation and biodegradation

Cold adapted microorganisms have been investigated for their potential use in the environmental remediation of oil-polluted mountainous soils (Margesin, 2000). Many components of petroleum hydrocarbons have been observed to possess biodegrading potential in Arctic, Alpine, and Antarctic soils, saltwater and Alaskan groundwater as well as in sub soils where temperatures persist up to 10°C (Margesin and Schinner, 1999; Margesin, 2000). *P. frigusceleri* MPC6 from Antarctica can convert a wide spectrum of chemicals, including phenols, benzene, toluene, and styrene (Orellana-Saez et al., 2019). The phenanthrene degradation potential of *P. frederiksbergensis* JAJ28T (DSM 13022 T) obtained from a gasification station in Frederiksberg, Denmark has been described (Andersen et al., 2000). *P. aeruginosa* holds significance in iron-rich sludge hydrothermal degradation and release phosphates (Yu et al., 2022) suggesting their surviving potential to a wide range of temperature.

*Pseudomonas* sp. MC1, which can thrive at temperatures ranging from 5 to 30°C, has been shown to decompose naphthalene. This property was attributed to the occurrence of thermolabile NahH enzyme which may be connected to the cold adaptation process of the bacteria with respect to the survival of MC1 strain on salicylate and naphthalene at low temperature conditions (Ahn et al., 2017). Degradation of polyhydroxyalkanoates (PHAs; Orellana-Saez et al., 2019), nitrobenzene, TNT (2,4,6-trinitrotoluene) were observed by *Pseudomonas* sp. MPC6, *P. frederiksbergensis* (NB-1; Cui et al., 2022) and *Pseudomonas* sp., isolated from Deception Island (Cabrera et al., 2022), respectively. While the bacterium was thought to be a good model strain for studying adaptation mechanisms in cold conditions, the possibility of its use as a bioremediator and biopolymer factory was also suggested (Orellana-Saez et al., 2019).

*Pseudomonas* species from the Himalaya are also being investigated for their ability to degrade chemicals present in medicines and personal care items (PPCP). For example, *Pseudomonas* sp. GBPI Hb5 and *P. palleroniana* GBPI 508 have been examined for caffeine and bisphenol A degradation, respectively (Thathola et al., 2021, 2022). These investigations will most likely have an impact on the bioremediation of PPCP from the contaminated settings.

## Industrial applications

The global demand for microorganisms and their products is constantly increasing in the last decade, from approximately USD 144 billion in 2010 to be an expected USD 303 billion by 2023 (McWilliams, 2018; Staff, 2018). Thus, they have been the prime focus for the industrially relevant materials such as enzymes, biofertilizers, etc. The products, prominently cryoenzymes extracted from psychrophiles have attained milestones in various disciplines*. Pseudomonas* species, isolated from cold environments, have been considered suitable for the production of cold-active enzymes (Li et al., 2014; Ganasen et al., 2016), for instance, *P. proteolytica* (GBPI_Hb61) isolated from cold Himalayan desert produce cold active lipase (Jain et al., 2017). *Pseudomonas* sp. LSK25 obtained from Signy Station, South Orkney Islands, maritime Antarctic reported for the production of recombinant lipase that is tolerant to organic solvents (Salwoom et al., 2019). Zhang and Zeng (2011) reported the identification of the amylase gene from the psychrotolerant *P. stutzeri* strain 7,193. Numerous other *Pseudomonas* species isolated from the regions like Indian Himalaya (Salwan et al., 2010; Jain and Pandey, 2016; Pandey et al., 2019; Jain et al., 2020), Ny-Alesund, Svalbard, Arctic (Srinivas et al., 2009), Humboldt Glacier, Mount Humboldt, Andean region of Venezuela (Ball et al., 2014) have been studied for production of industrially important enzymes such as amylases, proteases, RNA polymerase and lipases (Uma et al., 1999; Zhang and Zeng, 2011; Jain et al., 2017; Salwoom et al., 2019; Guo et al., 2021). Beside mountains, the oceans represent an untapped potential that exists in the microbiome to produce enzymes with distinctive properties. For example *P. marinensis* studied by Guo et al. (2021) possess two novel genes encoding for cold-active lipases. Such enzymes are also reported to survive and remain active at alkaline pH (Salwoom et al., 2019; Salwan et al., 2020).

Based on the global market data, the lipase demand was estimated as USD 420–430 million in 2018 with an approximate rise to USD 590–595 million by 2023 (Chandra et al., 2020). Certain intra and extracellular cold adapting enzymes namely phospholipase, protease, catalase, alcohol dehydrogenase, glycosyltransferase, alkaline phosphatase and translocase hold major applications in food and textile industries as well as biopharmaceuticals (Yang et al., 2010; Kumar et al., 2019). Farooq et al. (2022) suggested the efficiency of the gene amplification techniques employed to psychrotrophic *Pseudomonas* sp. CRBC14 isolated from the Himalayan glacier to induce the yield and purity of enzymes producing microorganisms. Being cold tolerant, these enzymes may also act as a food additive and preservative in food processing (Jin et al., 2022). Sah et al. (2021) highlighted various properties of *Pseudomonas* in the arena of bioaugmentation and biocontrol. Besides, they are also considered as good biocatalysts due to their strong catalytic activity which eliminates the need for heating operations that affects the efficiency, sustainability, and quality of industrial production (Santiago et al., 2016). As the enzymes must be used sequentially in processes and must be rendered inactive after serving their purpose, cold adapted enzymes are advantageous for molecular biosciences. When used in precise chemical synthesis, cold adapted esterases and lipases have been discovered to have a high degree of stereospecificity. Cold adapted enzymes’ stereospecificity may be helpful for the synthesis of chiral drugs (Cavicchioli et al., 2011). A possible substitute for the effective biofuel generation might be cold active enzymes (Wang et al., 2020). The serine alkaline proteases extracted from *Pseudomonas* spp., were demonstrated to remain active at a wide temperature and pH range (Zeng et al., 2003; Hao and Sun, 2015; Salwoom et al., 2019; Salwan et al., 2020), thus, behaves as efficient substrates for detergent preparation. Beside enzymes, the cold adapted microorganisms produce a range of bioactive compounds, pigments, polysaccharides, mainly with the purpose of their survival under low temperature stress; these compounds in turn have a range of applications in various industries (Sah et al., 2021).

Antarctic and Arctic environments are increasingly becoming the key habitats for the colonization of cold adapted microbial resources of biotechnological and industrial applications. Cold adapted species of *Pseudomonas* are receiving attention for their potential to produce exopolysaccharides that can be used as alternative source of commercial polysaccharides in biotechnology and various industries. A cold adapted Antarctic strain *Pseudomonas* sp. ID1 has been studied for the production of a new exopolysaccharide, mainly composed of glucose, fructose and galactose. Further, its biotechnological applications, as an emulsifier and cryoprotectant, have been highlighted; the emulsifying activity being higher as compared to the other commercial emulsifiers (Carrión et al., 2015). *Pseudomonas* sp. PAMC 28620, a psychrotrophic Arctic soil bacterium, has been reported for a distinct structural composition and metal removal and reduction potential in Arctic environment. The purified exopolysaccharide was recorded with remarkable flocculating and emulsification abilities leading to biotechnological applications such as metal adsorption (99%) potential showing the complexities and immobilization of metal ions and their further reduction to nanoparticles (Sathiyanarayanan et al., 2016). Various strategies such as optimization of culture media, secondary metabolites, physiological parameters, chemical derivatives etc., can be employed for enhanced production of the desired compound (Nair et al., 2021).

## Human health

The recent area of microbiome research has revealed the important role microorganisms are performing in our body; therefore, it is essential to explore microorganisms for the benefit of humankind. Microorganisms since their origin have played an important role in addressing the health issues right from the discovery of Penicillin which has save millions of lives. Species belonging to *Pseudomonas* were studied by Onbasli and Aslim (2008) for the synthesis of rhamnolipids, pyocins (bacteriocin), alginate, and exopolysaccharides. Rhamnolipids are also employed as biosurfactants from *Pseudomonas* sp. (Onbasli and Aslim, 2009; Braz et al., 2023), which have the potential to degrade oil and an efficient biocontrol molecule for long-term agricultural sustainability (Onlamool et al., 2022). Microorganisms may enhance the yield of secondary metabolites when a suitable substrate is added, for instance, *Pseudomonas* sp. was evaluated for an increase in rhamnolipid synthesis when supplemented with sugar beet molasses (Onbasli and Aslim, 2008; Braz et al., 2023). Further, it has been reported that the pyocins (F-and R-type) secreted by *Pseudomonas* sp. are a significant narrow spectrum antibiotic (Ge et al., 2020; Blasco et al., 2023). Domröse et al. (2015) worked on the development of PK/NRP hybrid compound which is similar to prodigiosin- a known immunosuppressant and anticancer agent. Methicillin-resistant *Staphylococcus aureus* (MRSA) can be treated alternatively using phenazines, a compound produced by *Pseudomonas* sp. (Cardozo et al., 2013). The same compound showed beneficial effects when combined with nanoparticles described by Cardozo et al., 2013. Afonso et al. (2022) examined the organocupric Fluopsin C (FlpC) isolated from *Pseudomonas* spp., with inhibitory activity against human and phytopathogens such as *Klebsiella pneumonia* (Navarro et al., 2019), *Xanthomonas citri* pv. *citri* (de Oliveira et al., 2011), *Xanthomonas citri* subsp*. citri* (de Oliveira et al., 2016), *Xanthomonas axonopodis* (Lopes et al., 2012) etc. Tyrosol was studied by Allouche et al. (2004) and proven to boost the production of hydroxytyrosol, a powerful antioxidant that can also be generated by immobilization (Bouallagui and Sayadi, 2006). *P. putida* was observed for *de novo* production of geranic acid (terpenoid), carotenoids, polyketides, *mcl-* PHA (polyhydroxyalkanoates) with reported antibiotic potential and uses as fragrance and flavor component (Weimer et al., 2020).

*Pseudomonas* is also known as a source of exopolysaccharides such as pellicle loci (Pel), polysaccharide synthesis loci (Psl) and alginate (Franklin et al., 2011), which are important structural elements of biofilm formation and are believed to be essential for a variety of purposes such as coherence, adherence, enzyme binding, resistance to abiotic stress, and antimicrobial agents (Jennings et al., 2015; Heredia-Ponce et al., 2021; Fleming et al., 2022). Psl and Pel are also reported for the synthesis of mannose and glucose-rich matrix, respectively (Friedman and Kolter, 2004). These characteristic features of the biofilm matrix of *Pseudomonas* spp. may help in the early diagnosis of *Pseudomonas* related infections. Alginate is a major component with many medical applications, for instance, advanced wound care dressings employ alginate hydrogels to absorb fluid creating a moist environment that lowers the risk of infection (Paul and Sharma, 2004) and a popular bio-ink used to create biocompatible, non-immunogenic frameworks for the 3D printing of tissues and organs (Lee and Mooney, 2012).

Antibiotic resistance in *Pseudomonas* is a rising problem in both clinical and community settings, limiting the efficacy of many traditional treatment approaches. *P. aeruginosa* strains possess resistance-nodulation-division efflux pumps that physically sequester incoming drugs (Hwang and Yoon, 2019) and disturb the action mechanism of the drugs; also the genetic mutations or horizontal gene transfer exhibit intrinsic antibiotic resistance (Pestrak et al., 2019). Furthermore, biofilms serve as a barrier against antibiotic penetration. In this context, researchers have developed recombinants formed by the omission of virulence genes present within the *Pseudomonas* genome (Valentine et al., 2020; Farooq et al., 2022). Rezzoagli et al. (2020) identified antibiotic-antivirulence combinations as a potentially powerful tool to efficiently treat infections of nosocomial pathogens caused by *P*. *aeruginosa.* Horcajada et al. (2019) reported ceftolozane-tazobactam combination to possess significant antimicrobial activity against an extensively drug-resistant and multidrug-resistant *Pseudomonas*.

## Cosmetics

Over the last few decades, it has been observed that the cosmetic industries are developing interest in the bio-based products. Due to the rising health issues from chemical based products, various developed and developing countries are now focusing on the microbe based products for health benefits. Several microorganism-derived products have shown huge potential in cosmetic industry. Specifically talking about *Pseudomonas*, alginate produced by *P. aeruginosa* possesses good water holding ability and is thus utilized in the manufacture of skin moisturizer and gelling agents (Valentine et al., 2020). *Pseudomonas* spp., however, are well known opportunistic microorganisms reported worldwide for causing various fatal infections. Thus, the concept of recombinants development plays a significant role and allows the physiology and genetic makeup of the organism. The *in-vivo* observations noted by Valentine et al. (2020) supported the mentioned fact. In this study, five important pathogenicity genes were omitted from the *P. aeruginosa* genome using a homologous recombination technique that decreased the systemic virulence and also enhanced the yield of alginate.

A SEM and TEM analysis suggested the presence of melanin in *P. koreensis* UIS 19 with high tyrosinase activity. The strain demonstrated a promising sun protection factor (SPF) value of more than 60 for 20 mg/mL and more than 92% effective in scavenging DPPH radicals (Eskandari and Etemadifar, 2021). *Pseudomonas* sp., is known to produce another important compound, Vanillin with a wide application in cosmetic industry. It is basically used to provide a pleasant aroma and flavor while also enhances the texture of the products. Wang et al. (2021) developed a recombinant isoeugenol enzyme extracted from *P. nitroreducens* Jin1 by optimizing the culture conditions in order to obtain vanillin, which further increased the yield by 82% with more than 95% purity.

## Conclusion and future prospects

Cold adapted microorganisms, inhabiting low temperature environments, address the growing concerns related to ecosystem protection in the present climate change scenario. In this context, Pseudomonads are seen as one of the most metabolically diversified bacterial colonizers of cold environments. Their capacity to grow in a wide range of temperatures (as psychrophiles or psychrotolerants) and pH (extreme acidic to alkaline) makes them ideal candidates to withstand the extreme industrial processes. Their abilities to produce bioactive chemicals, enzymes, and pigments allow them for their utilization in a variety of ecological and biotechnological applications. Utilizing the recently developed technologies and methodologies for cultivation of majority of the microorganisms, which are yet to be cultured, will be an important step to explore the biodiversity and metabolic potentials of *Pseudomonas.* Given their multifarious uses, conservation of microorganism from such ecosystems in National and International Microbial Resource Centers is essential for a sustainable future.

## Author contributions

AP and MC: conceptualization and draft preparation. AK and MC: figures and tables. AP and AS: reviewing and editing. All authors contributed to the article and approved the submitted version.

## Conflict of interest

The authors declare that the research was conducted in the absence of any commercial or financial relationships that could be construed as a potential conflict of interest.

## Publisher’s note

All claims expressed in this article are solely those of the authors and do not necessarily represent those of their affiliated organizations, or those of the publisher, the editors and the reviewers. Any product that may be evaluated in this article, or claim that may be made by its manufacturer, is not guaranteed or endorsed by the publisher.
